# Comment on ‘Adherence to Physical Activity and Incident Mobility Disability in Older Adults With Mobility Limitations’ by Álvarez‐Bustos et al.

**DOI:** 10.1002/jcsm.70068

**Published:** 2025-09-23

**Authors:** Bo Zhang, Haijun Zhang

**Affiliations:** ^1^ Department of Orthopedics Ningbo No. 2 Hospital Ningbo China

We read with considerable interest the valuable contribution of Álvarez‐Bustos et al., who demonstrated that older adults in a frailty prevention trial attending more weekly exercise sessions experienced a lower risk of mobility disability [1]. The authors' work advances our understanding of physical activity (PA) patterns in this vulnerable population. However, we would like to offer some thoughtful considerations regarding the measurement of exercise adherence, which may enhance the translation of such important findings to clinical practice.

The study's approach to defining adherence exclusively through session frequency (below/meeting/above the ACSM guidelines) represents a pragmatic yet potentially limited perspective on exercise dosing. Although intuitively appealing, this frequency‐based classification may inadvertently overlook the nuanced reality of exercise delivery. To illustrate this complexity, consider two participants who each attended three sessions weekly: One may engage in brisk walking for 30 min at moderate intensity, while the other participates in light‐effort strolling for 10 min. Despite identical frequency classifications, these dramatically different exercise experiences would predictably yield distinct physiological adaptations and functional outcomes. This scenario suggests that session counting, while convenient, may lead to misclassification where differences in intensity, duration and exercise modality become obscured.

Furthermore, the frequency‐only approach raises important questions regarding reverse causality. Participants with better baseline health or higher fitness levels may naturally possess a greater capacity to attend frequent sessions, potentially creating a scenario where ‘high adherence’ primarily identifies an inherently fitter subgroup rather than reflecting true exercise dose–response relationships [2]. This consideration becomes particularly relevant when interpreting observed benefits in the context of mobility disability prevention.

Recent evidence from objective measurement studies has provided compelling support for more comprehensive adherence assessment approaches. A notable 9‐year Japanese cohort study involving participants with a median age of 73 years demonstrated that accelerometer‐measured moderate‐to‐vigorous physical activity (MVPA) and sedentary time predicted incident functional disability with greater precision than simple activity counts [3]. The study revealed that replacing merely 10 min of sedentary time with 10 min of MVPA was associated with a 12% reduction in future disability risk, while substituting light‐intensity activity showed no significant benefit [3]. This finding elegantly illustrates how exercise intensity and actual minutes of moderate‐pace activity have substantial prognostic value, suggesting that relatively small increases in vigorous effort can yield meaningful clinical effects.

The LIFE trial further reinforced these observations by demonstrating dose‐dependent relationships between objectively monitored activity and mobility outcomes [4]. Participants in the highest quartile of increased MVPA experienced substantially lower disability rates than those in the lowest quartile, emphasizing that both the quantity and intensity of movement predicted functional outcomes [5, 6]. More recently, sophisticated machine learning analyses of accelerometer data have identified 11 combined features, including overall volume, bout duration, intensity distribution and frequency patterns, which significantly improve mortality risk prediction beyond traditional factors [7]. These composite metrics, rather than simple weekly frequency counts, capture the most clinically relevant signals for adverse outcomes.

Given these insights, we propose several feasible strategies to refine adherence measurements in future trials. First, incorporating wearable sensors, such as accelerometers, pedometers and heart‐rate monitors, can provide comprehensive dose profiles, including total MVPA minutes, step counts, energy expenditure and heart‐rate zone distributions [8, 9]. A recent heart failure exercise trial exemplified this approach by defining adherence as achieving at least 120 min weekly at 40%–80% heart rate reserve, verified through wearable monitoring [10]. This methodology ensured that each session met moderate intensity thresholds.

Second, developing composite adherence indices that integrate attendance with intensity and duration metrics could provide more nuanced dosing information [11]. Building upon emerging methodological approaches, researchers might construct multicomponent scores combining total MVPA minutes, exercise bout length, and session‐specific ratings of perceived exertion to quantify comprehensive exercise ‘dose’ [7]. Third, applying sophisticated analytical approaches, such as isotemporal substitution modelling in data analysis, can clarify how incremental changes in specific exercise parameters affect mobility outcomes [3, 12].

Practically, clinical trials could expand beyond session attendance recording to include participants' accelerometry or heart rate monitoring during both supervised and home‐based exercise sessions. This approach captures the intensity and volume dimensions that session counting alone cannot provide, yielding richer insights into the true exercise dose–response relationships underlying mobility preservation.

We deeply appreciate the important contributions of Álvarez‐Bustos et al. in this field, suggesting that future investigations might benefit from more comprehensive adherence assessment approaches. By integrating wearable‐derived metrics and composite scoring systems, researchers can generate more insights into PA adherence and mobility outcomes. We encourage continued refinement of adherence definitions in multimodal interventions, incorporating both attendance patterns and quantitative measures, such as mean MVPA minutes or session‐specific intensity ratings. Such approaches would enable clinical recommendations to address not only ‘how often’ but also ‘how much and how intensely’ older adults should exercise to effectively prevent mobility disability.

## Ethics Statement

The authors have nothing to report.

## Conflicts of Interest

The authors declare no conflicts of interest.

## Data Availability

No new data were generated or analysed in support of this work.
